# Supplementary material for: Discovery of an intermediate nematic state in a bilayer kagome metal ScV6Sn6
Source: Nat Commun. 2025 Aug 23;16:7867. doi: 10.1038/s41467-025-63294-5 (PMC12375086; doi:10.1038/s41467-025-63294-5)
Supplement: Supplementary file 1 — Supplementary Information [file 41467_2025_63294_MOESM1_ESM.pdf]

Supplementary Information for

Discovery of an Intermediate Nematic State in a Bilayer Kagome Metal ScV_6Sn_6

Camron Farhang,[1] William R. Meier,[2] Weihang Lu,[1] Jiangxu Li,[3] Yudong Wu,[1] Shirin Mozaffari,[2] Richa P. Madhogaria,[2] Yang Zhang,[3,4] David Mandrus,[2,5] Jing Xia[1,*]

[1] Department of Physics and Astronomy, University of California, Irvine, Irvine, CA 92697, USA

[2] Department of Materials Sciences and Engineering, University of Tennessee-Knoxville, Knoxville, Tennessee 37996, USA

[3] Department of Physics and Astronomy, University of Tennessee-Knoxville, Knoxville, Tennessee 37996, USA

[4] Min H. Kao Department of Electrical Engineering and Computer Science, University of Tennessee-Knoxville, Knoxville, Tennessee 37996, USA

[5] Materials Science and Technology Division, Oak Ridge National Laboratory, Oak Ridge, TN 37831, USA

*Correspondence: xia.jing@uci.edu

Contents:

Supplementary figures 1-5 pages 2-6

Structure information used in DFT calculation page 7

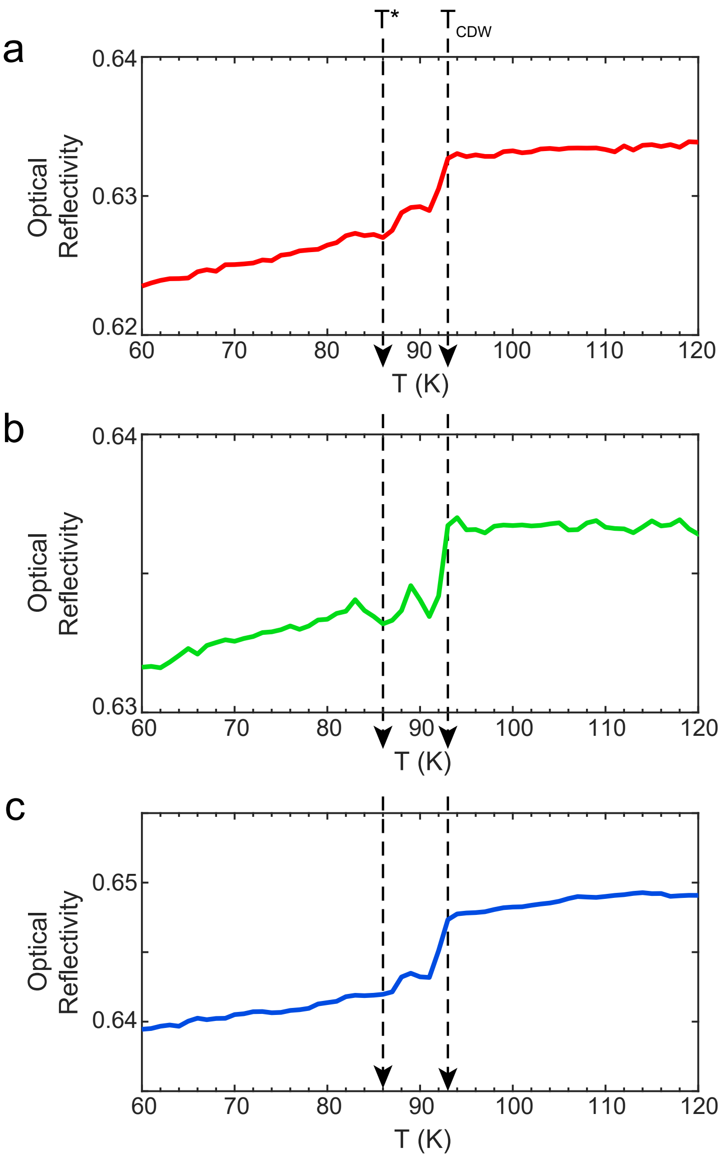

Supplementary Figure 1. Temperature-dependent optical reflectivity (1.55 μm) measured at different locations. a-c, Consistently at all measured locations, we observe a ~0.8% drop in optical reflectivity when the sample is cooled through T_{CDW}, and another ~0.3% drop when cooling through T^*. These findings demonstrate that the observed double phase transitions at T_{CDW} and T^* are not attributable to macroscopically separated volumes in the sample with two distinct CDW transition temperatures, but rather are localized intrinsic phenomena. The absolute values of optical reflectivity exhibit a spatial variation of 0.015, indicating a small 2% inhomogeneity across the entire sample.

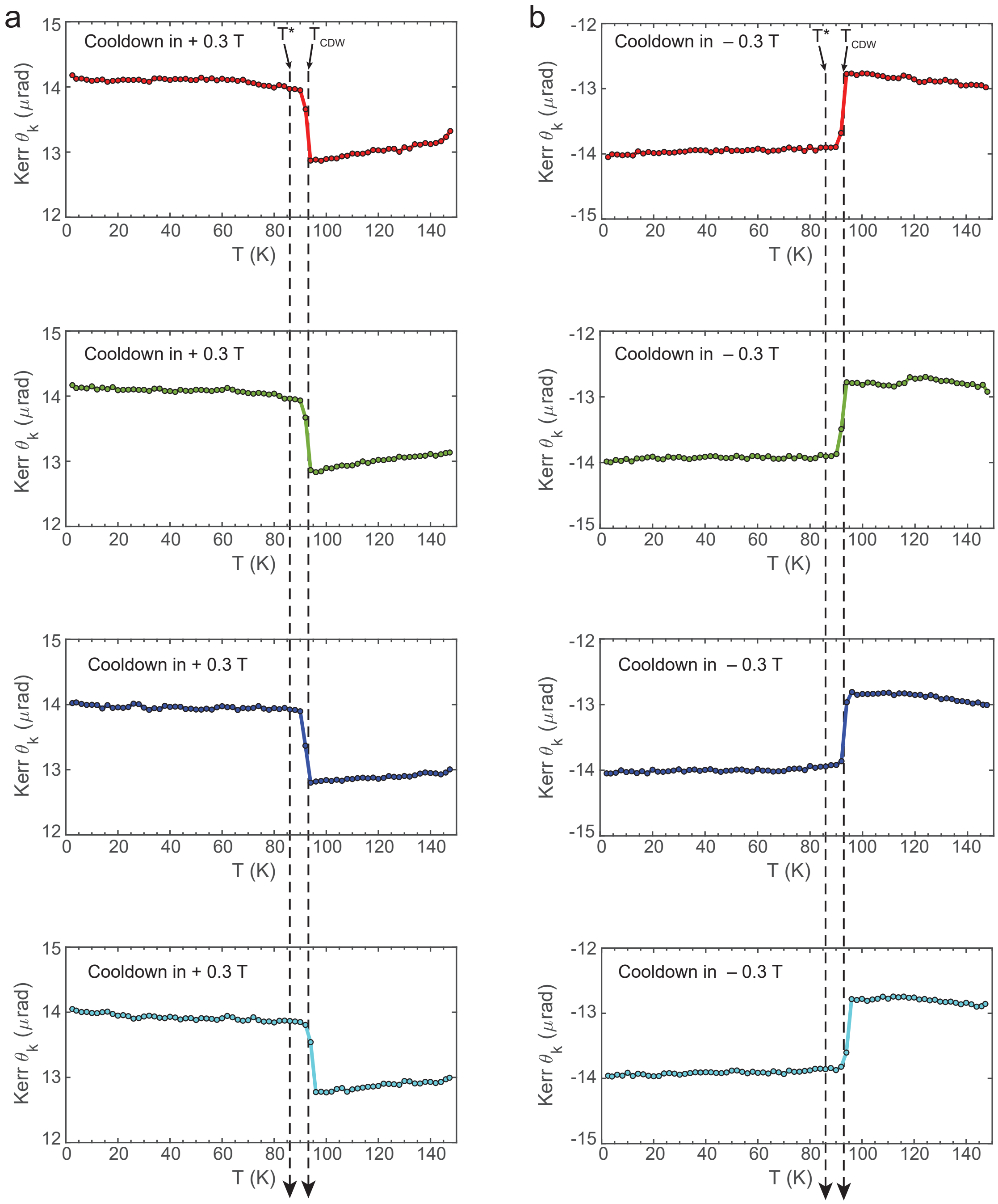

Supplementary Figure 2. MOKE θ_K measured during field cooldowns. a, θ_K during **B** = + 0.3 T field cooldowns. **b**, θ_K during **B** = − 0.3 T field cooldowns. The results show a paramagnetic MOKE response with a sharp increase below T_{CDW}, but no change across T^*, indicating the T^* transition is not coupled to the magnetic degree of freedom.

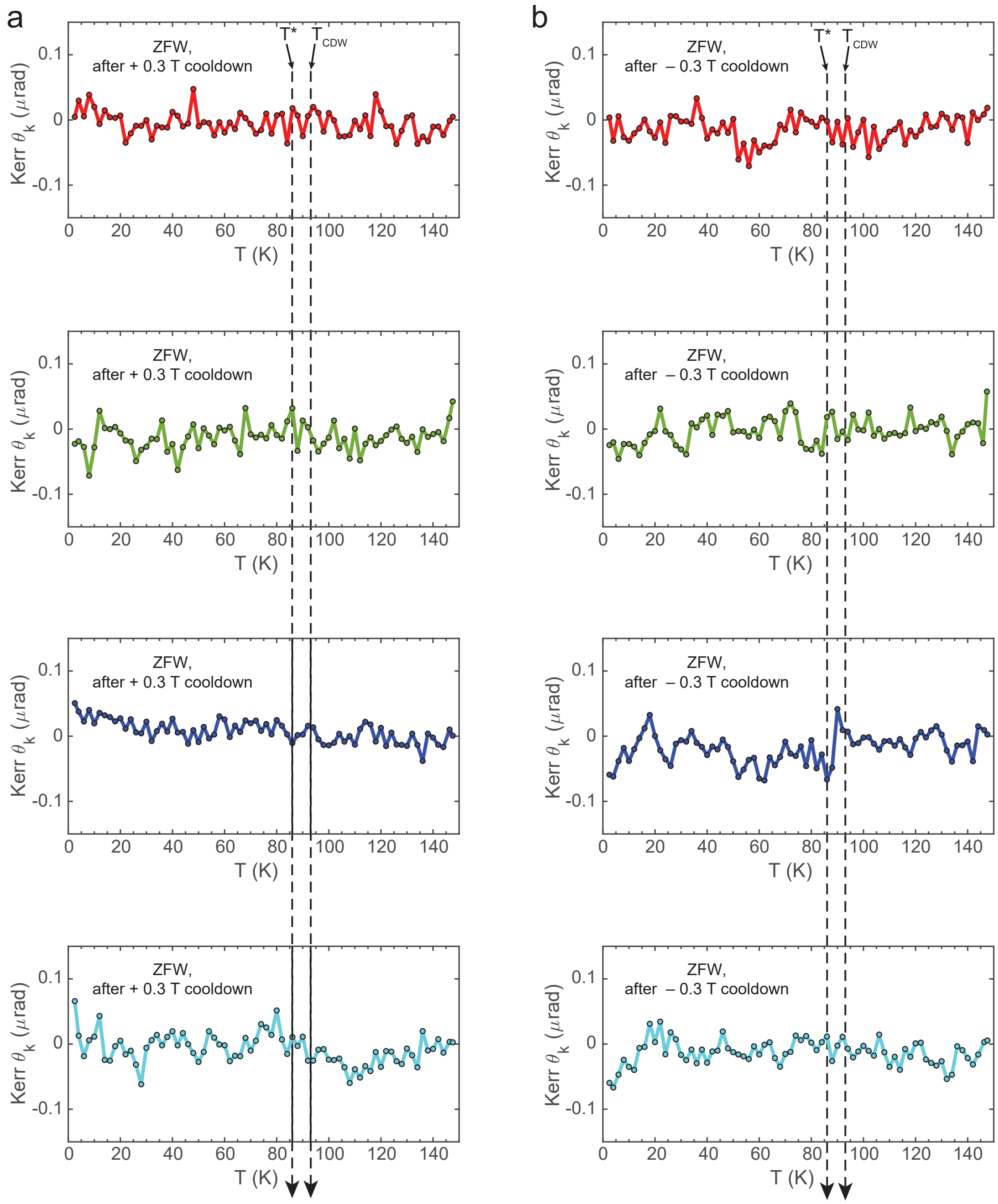

Supplementary Figure 3. Spontaneous MOKE θ_K measured during zero-field warmups (ZFW). a, ZFW after **B** = + 0.3 T field cooldowns. **b,** ZFW after **B** = – 0.3 T field cooldowns. No discernable onset of spontaneous θ_K was found at either T_{CDW} or T^* with $\pm 30\ nrad$ uncertainty, showing no evidence for TRSB.

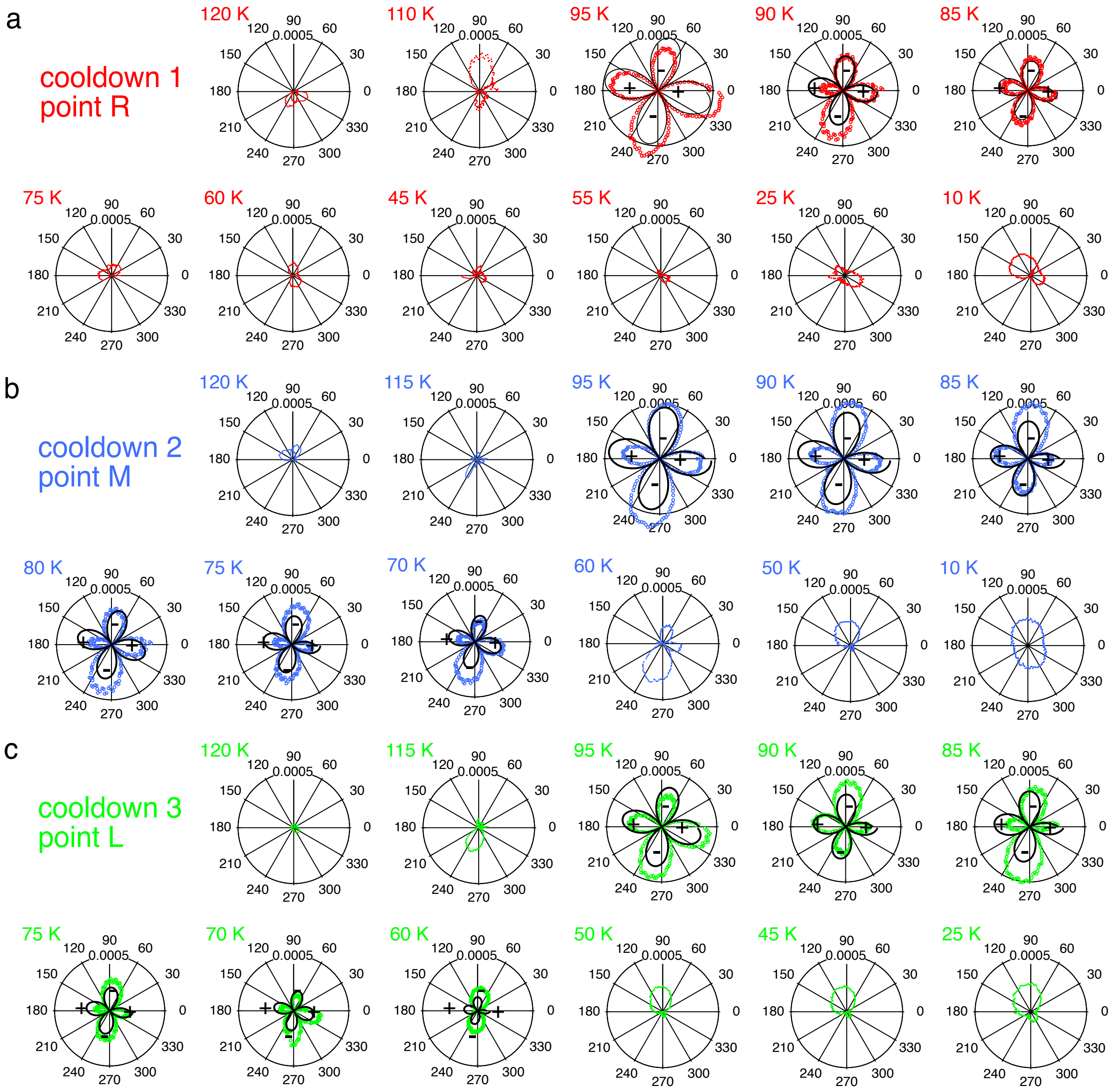

Supplementary Figure 4. Polar plots of optical polarization rotation measured at various locations on the ab-plane and at various temperatures. a-c, The development of four-leaf clover pattern with alternating "+" and "-" signs indicates intrinsic ab-plane anisotropy. The full scale of the polar plots is $0.0005\,rad$. While most of the anisotropy is observed between T_{CDW} and T^*, at points M and L the anisotropy persists to 70 K and 60 K respectively.

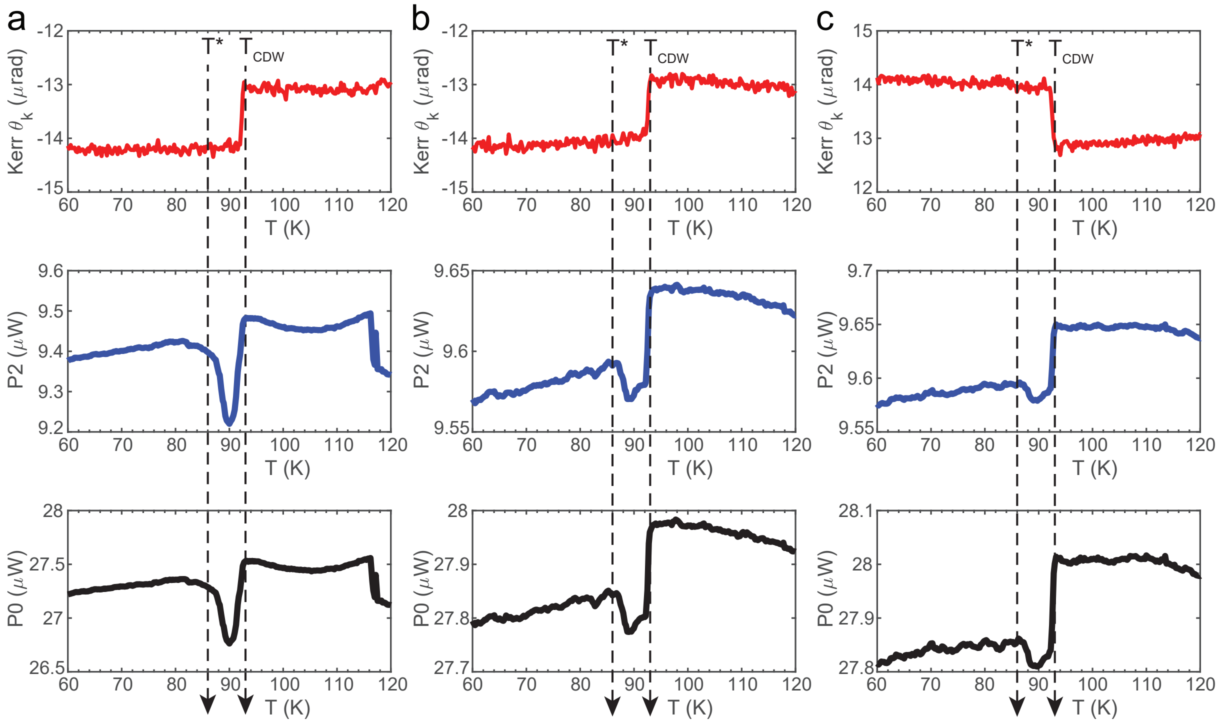

Supplementary Figure 5. $\boldsymbol{\theta_K}$, $\boldsymbol{P_2}$, and $\boldsymbol{P_0}$ simultaneously recorded in Sagnac interferometer measurements at various locations. a-b, B $= -$ 0.3 T cooldown. c, B $= +$ 0.3 T cooldown. As explained in Methods, the measured θ_K is the MOKE signal, which is independent of sample reflectivity or depolarization effects. P_2 and P_0 on the other hand record the part of reflected optical power that is not depolarized by the sample surface. The depolarization effect here arises from the ab-plane anisotropy in ScV_6Sn_6. Profoundly, cooling from T_{CDW} to T^*, the reflectivity (Supplementary Fig.1) shows a drop-plateau-drop while P_2 and P_0 exabit a single pronounced dip, revealing a large depolarization effect between T_{CDW} and T^*, which is consistent with the observed transport and optical anisotropy. Further, between different locations, we observe a difference in the size and the lower temperature bound of the dip in P_2 and P_0. This inhomogeneity echoes that observed in the polarization rotation measurements presented in Supplementary Fig.4.

Structure information used in DFT calculation:

Structure information used in DFT calculations is included in plain text format as Supplementary Datasets. "ScV6Sn6_280K_dft_prim.cif" is for the high-temperature (280 K) phase. "ScV6Sn6_50K_dft_prim.cif" is for the low-temperature CDW (50 K) phase.
